# Electronic smart-hub based intervention during COVID-19 in a rural Psychiatry of Old Age service in North-West Ireland

**DOI:** 10.1192/j.eurpsy.2022.1679

**Published:** 2022-09-01

**Authors:** S. Patel, A. Gannon, M. Cryan, C. Dolan, C. Mcdonald, C. Houghton, G. Mccarthy

**Affiliations:** 1 University Hospital Galway, Psychiatry, Galway, Ireland; 2 NUI Galway, School Of Medicine, Galway, Ireland; 3 Sligo Leitrim Mental Health Services, St. Columba’s Hospital, , Psychiatry, Sligo, Ireland; 4 Sligo Leitrim Mental Health Services, St. Columba’s Hospital, , Psychiatry, Sligo, Ireland; 5 NUI Galway, School Of Nursing And Midwifery, Galway, Ireland

**Keywords:** Covid-19, smart hub, Older Adults, technology

## Abstract

**Introduction:**

The COVID-19 pandemic caused significant disruptions in services and necessitated innovation to continue care provision to the vulnerable population of older adults with psychiatric needs.

**Objectives:**

The objective of this study was to examine the experiences of staff and patients using a hands-free electronic smart-hub (eSMART hub) intervention to keep patients connected with psychiatry of old age following COVID-19 restrictions.

**Methods:**

A risk stratification register was created of all patients known to the Psychiatry of Old Age service in the North-West of Ireland to identify those at highest risk of relapse. These patients were offered a smart-hub with remote communication and personal assistant technology to be installed into their homes. Smart-hubs were also installed in the team base to facilitate direct device to device communication. Semi-structured qualitative interviews were conducted with 10 staff members and 15 patients at 6-12 months following the installation of the smart-hubs.

**Results:**

The smart-hubs were utilized by the POA team to offer remote interventions over video including clinician reviews, regular contact with key workers and day-hospital based therapeutic interventions such as anxiety management groups and OT led physical exercises. Patients also used the personal assistant aspect of the hub to attend to personal hobbies such as accessing music and radio. Positive feedback related to companionship during isolation and connectivity to services. Negative feedback was mainly related to technology, particularly internet access and narrow scope of communication abilities.

**Conclusions:**

Electronic smart-hub devices may offer an acceptable avenue for remote intervention and communication for isolated high-risk older persons.

**Disclosure:**

The smart hub devices used in this study were donated by Amazon. However, the company was not involved in any other aspect of the study and the researchers have no significant financial interest, consultancy or other relationship with products, manufactur

